# Overexpression of the Wheat Expansin Gene *TaEXPA2* Improved Seed Production and Drought Tolerance in Transgenic Tobacco Plants

**DOI:** 10.1371/journal.pone.0153494

**Published:** 2016-04-13

**Authors:** Yanhui Chen, Yangyang Han, Meng Zhang, Shan Zhou, Xiangzhu Kong, Wei Wang

**Affiliations:** 1 State Key Laboratory of Crop Biology, Shandong Key Laboratory of Crop Biology, College of Life Sciences, Shandong Agricultural University, Tai’an, Shandong, 271018, P. R. China; 2 Plastic Surgery Institute of Weifang Medical University, Weifang, Shandong, 261041, P. R. China; Nanjing Agricultural University, CHINA

## Abstract

Expansins are cell wall proteins that are grouped into two main families, α-expansins and β-expansins, and they are implicated in the control of cell extension via the disruption of hydrogen bonds between cellulose and matrix glucans. *TaEXPA2* is an α-expansin gene identified in wheat. Based on putative cis-regulatory elements in the *TaEXPA2* promoter sequence and the expression pattern induced when polyethylene glycol (PEG) is used to mimic water stress, we hypothesized that *TaEXPA2* is involved in plant drought tolerance and plant development. Through transient expression of *35S*::*TaEXPA2-GFP* in onion epidermal cells, TaEXPA2 was localized to the cell wall. Constitutive expression of *TaEXPA2* in tobacco improved seed production by increasing capsule number, not seed size, without having any effect on plant growth patterns. The transgenic tobacco exhibited a significantly greater tolerance to water-deficiency stress than did wild-type (WT) plants. We found that under drought stress, the transgenic plants maintained a better water status. The accumulated content of osmotic adjustment substances, such as proline, in *TaEXPA2* transgenic plants was greater than that in WT plants. Transgenic plants also displayed greater antioxidative competence as indicated by their lower malondialdehyde (MDA) content, relative electrical conductivity, and reactive oxygen species (ROS) accumulation than did WT plants. This result suggests that the transgenic plants suffer less damage from ROS under drought conditions. The activities of some antioxidant enzymes as well as expression levels of several genes encoding key antioxidant enzymes were higher in the transgenic plants than in the WT plants under drought stress. Collectively, our results suggest that ectopic expression of the wheat expansin gene *TaEXPA2* improves seed production and drought tolerance in transgenic tobacco plants.

## Introduction

The expansins are a multigene family that includes the α-expansin (EXPA), β-expansin (EXPB), expansin-like A, and expansin-like B subfamilies. The amino acid sequences of the expansin proteins in these four subfamilies exhibit only 20–40% identity. EXPA and EXPB, the two major groups, share several conserved motifs and likely have similar, but different, functions [1]. Analysis of the gene structures and predicted amino acid sequences of rice and *Arabidopsis* expansins indicates that the α- and β-expansin genes evolved from one ancestral gene. The exon/intron organization is unchanged, but the length of each intron and the number of introns differs among the individual genes [2–3].

It is believed that the functions of the α- and β-expansin genes are similar and different in a number of ways. For instance, two constitutively expressed *Arabidopsis* expansin genes, *AtEXP3* and *AtEXP-β1*, representing the α-expansin and β-expansin subfamilies, respectively, changed transgenic *Arabidopsis* leaf development: in the former, transgenic *Arabidopsis* leaf area was larger than wild-type (WT) leaf area, whereas in the latter, transgenic *Arabidopsis* plants showed longer petiole lengths compared to WT plants. Salt sensitivity, by contrast, increased in both transgenic *Arabidopsis* lines, but the mechanisms of this increase were different, as seen by the differing transcription levels of two stress-associated genes, *COR15a* and *KIN1*, hours after salt treatment [4].

Adverse environmental conditions, such as drought and salt stress, affect plant growth and development. As one of the principal natural limitations on plant productivity, low water potential caused by insufficient soil water causes large economic losses in many regions [5]. Water deficits restrain plant growth by delaying both cell extension and cell division. During cell division and elongation, the cell wall must be extended and it must form a cellulose-hemicellulose network that is linked via hemicellulosic tethers [6]. Expansins have been implicated in the control of plant growth via the loosening of the extracellular matrix in a pH-dependent manner [7–8]. There is increasing evidence for the involvement of expansins in the responses of various plant species to water stress. For example, increased expansin activity was found to be involved in maintaining the growth of maize primary roots at a low water potential [9–10]. The resurrection plant *Craterostigma plantagineum* has been used as a model for investigating desiccation tolerance at the molecular level, and cell wall extensibility in C. *plantagineum* leaves is known to increase during drying. At the same time, expansin activity and the expression levels of expansin genes in C. *plantagineum* increased during the process of dehydration and rehydration [11]. The expression of a rose expansin gene, *RhEXPA4*, is remarkably up-regulated in rose petals after dehydration [12], and it confers salt and drought tolerance to transgenic *Arabidopsis* [13]. *GmEXPB2* is a root β-expansin gene that participates in root system architecture responses to several abiotic stresses, such as Fe, P, and water deficiency [14].

Wheat is cultivated as one of the most significant food crops worldwide, and drought stress is an acute environmental condition that can severely block the growth and productivity of wheat [15]. It has been estimated that there are at least 30 α-expansin and 65 β-expansin genes in wheat [16]. Our previous study indicated that wheat expansin expression responds to water stress [17–18]. The ectopic expression of *TaEXPB23*, a wheat β-expansin gene, improved the drought tolerance and changed the phenotype of transgenic tobacco [19–20]. In this study, to discover the similarities and differences in the functions of α- and β-expansins, we selected a wheat α-expansin gene, *TaEXPA2* (GenBank ID: AAT94292.1), and investigated its involvement in plant drought tolerance and development.

## Materials and Methods

### Plant materials, growth conditions, and water stress treatments

Wheat seeds (*Triticum aestivum* L.) were germinated at 25°C in the dark on absorbent cotton gauze drenched in sterile water on an enameled dish. The seedlings were then spread out uniformly in buckets to create hydroponic cultures in an illuminated incubation chamber (GXZ-500C, Jiangnan, China) under a relative humidity of 75%, a photosynthetically active radiation level about 300 μmol m^-2^ s^-1^, and a 16/8 h light/dark cycle at 25°C. The 1/2 Hoagland’s nutrient solution was renewed every 2 days. After 7 days, when two real leaves were fully expanded, 20% PEG, 150 mM NaCl, 2 mM ABA, 50 mM GA, 10 mM MeJA, or 10 mM SA was added to the nutrient solution to initiate the experimental treatments. Seven-day-old wheat seedlings grown in 1/2 Hoagland’s nutrient solution without any supplementation were used as a control group.

The tobacco seedlings used (*Nicotiana tabacum* ‘NC89’) were grown from seeds that were surface sterilized with 70% ethanol for 1 min, 4% NaClO for 8 min, and then washed four times with sterile deionized water. The seeds were germinated in Murashige-Skoog (MS) media in a controlled environment at 25°C under a photoperiod of 16/8 h (light/dark).

To measure the germination rates of transgenic tobacco lines under water stress, 50 seeds from transgenic and WT lines were surface-sterilized and sown in triplicate on MS plates supplemented with mannitol at concentrations of 0 mM, 100 mM, and 200 mM. The plates were placed under a photoperiod of 16/8 h (light/dark) at 25°C, and the germination rates were calculated after 10 days.

To induce water stress in seedlings grown on MS media, 7-day-old tobacco seedlings were transplanted to new MS media with a different concentration of mannitol (0 mM, 100 mM, or 200 mM). The primary root length and lateral root length were tracked and analyzed statistically 20 days after treatment.

For the dehydration treatment, 7-day-old tobacco seedlings grown on vermiculite were placed in an illuminated incubation chamber (GXZ-500C, Jiangnan, China) with photosynthetically active radiation of about 300 μmol m^-2^ s^-1^, a relative humidity of 75%, and a 16/8 h light/dark cycle at 25°C. They were left without irrigation for 7 days and were then re-watered for 2 days. Ten seedlings were planted in each pot, and the survival rate was recorded after re-watering.

To determine the drought stress tolerance of the grown tobacco plants, transgenic and WT plants grown on vermiculite for 50 days under normal growing conditions were transferred to a climate-controlled greenhouse and received no watering for another 21 days. The greenhouse was controlled at 25°C with a 16/8 h light/dark cycle (400–600 μmol photons m^-2^ s^-1^) and a relative humidity of 70–80%. A control group was watered normally. The fully expanded leaves were used to calculate the physiological indexes. Phenotypic indexes, such as plant height, leaves, capsules, and seeds, were observed and recorded at intervals during plant growth and development.

### Cloning and assay of cis-regulatory elements in the promoter region of *TaEXPA2*

Based on the expansin gene sequence, we designed custom reverse gene primers (*GSP1*, *GSP2*, *GSP3*). The isolation of the *TaEXPA2* gene promoter was achieved using the thermal asymmetric interlaced PCR (TAIL-PCR) technique. From two successful TAIL-PCR runs, we obtained a 1073-bp region upstream of the translation initiation codon (GenBank ID: KP729264). The cis-regulatory elements in the promoter sequence of *TaEXPA2* were analyzed using PlantCARE (http://bioinformatics.psb.ugent.be/webtools/plantcare/html) and PLACE (http://www.Dna.affrc.go.Jp).

### Subcellular localization of the TaEXPA2 protein

The open reading frame (ORF) of *TaEXPA2*, minus the termination codon, was acquired by PCR amplification utilizing the specific primers *GFP1* and *GFP2*. The purified PCR products were linked into a pMD18-T simple vector (TaKaRa, Japan) and digested by BamH 1 and Sal 1. The fragments were fused to the N-terminus of the green fluorescent protein (GFP) expression vector PBI121 under the control of a CaMV 35S promoter (*35S*::*TaEXPA2-GFP*). The vector *35S*::*GFP* was used as control. The recombinant pBI121 plasmids were introduced into *Agrobacterium tumefaciens* strain LBA4404, which was then used to transform onion (*Allium cepa*) epidermal cells. The onion epidermal tissues were cultivated on MS plates under dark conditions at 28°C for 24 h after transformation. A confocal microscope (Olympus, Tokyo, Japan) was used to observe the transformed onion cells.

### Generation of *TaEXPA2*-overexpressing tobacco lines and quantitative real-time PCR analysis

Total RNA was extracted from wheat leaves with Trizol reagent (TaKaRa, Japan) according to the manufacturer’s instructions. Total RNA (2 μg) was reverse-transcribed into cDNA using the RevertAid First Strand cDNA Synthesis Kit (Fermentas, USA). Full-length cDNA of *TaEXPA2* (GenBank accession number: AAT94292.1) was obtained by PCR using specific primers (named: *A2-F*, *A2-R*), and then this fragment was joined to pBI121 under the control of the CaMV 35S promoter. The recombinant plasmid *35S*::*TaEXPA2* was introduced into the *Agrobacterium tumefaciens* strain LBA4404, and then through leaf disc transformation the plasmid was mediated into WT plants. Independent transgenic lines were selected by kanamycin resistance.

To assay gene expression, quantitative real-time PCR (qRT-PCR) analysis was performed with the RNA samples prepared at the indicated time points after treatments. The qRT-PCR reactions were performed in a Bio-RAD MyiQ^™^ Real-time PCR Detection System (Bio-Rad, USA) using TransStart Top Green qPCR SuperMix (TransGen, China) according to the manufacturer’s instructions. Each reaction system (20 μl) contained 10 μl of 2× real-time PCR mix (containing SYBR Green I), 1 μl of PCR forward or reverse primer, and adequately attenuated cDNA. The PCR temperature cycling conditions were 95°C for 30 s followed by 45 cycles of 95°C for 15 s, 58°C for 30 s, and 72°C for 15 s. The α*-tubulin* gene was used as an internal reference for all of the qRT-PCR analyses. Each treatment was repeated at least three times independently. Using the delta-delta Ct method, the relative expression levels were calculated.

To assay the response of *TaEXPA2* expression in wheat under the different treatments described above, qRT-PCR analysis was performed using gene-specific primers (named: *RTA2-F*, *RTA2-R*) and *NtACTIN* as an internal reference.

To assay the expression of *TaEXPA2* in the WT and five independent transgenic plants, qRT-PCR was performed with the RNA samples extracted from 4-week-old seedlings.

To assay the expression of antioxidant related genes, qRT-PCR analysis was performed with RNA samples isolated from leaves harvested after treatment with or without drought stress. The primers used were the same as those in Wu et al. [21].

The primer sequences used in this experiment are provided in S1 Table.

### Expansin activity and immunoblot analysis of TaEXPA2 protein abundance in the cell walls of transgenic tobacco

The prepared cell wall protein from transgenic tobacco leaves was extracted as described by Gao et al. [17]. Expansin activity was assessed following Zhou et al. [22]. Proteins were separated by SDS-PAGE on a 12% gel and transferred onto a polyvinylidene fluoride (PVDF) membrane (Millipore, Saint-Quentin, France). Protein accumulation was then tested with an antibody against the TaEXPA2 protein, custom-made by GenScript (Nanjing, Jiangsu, P.R. China).

### Measurements of chlorophyll, proline content, leaf water loss kinetics, and relative water content

Fresh leaves (0.1 g) were ground smoothly with 95% ethanol. After centrifugation, the extract was examined with a spectrophotometer at three different peak wavelength (645, 663, and 652 nm). The content of chlorophyll was calculated by the method described by Knudson et al. [23].

The proline content was assayed according to Dörffling et al. [24].

The kinetics of leaf water loss rate was conducted according to Li et al. [25]. Relative water content (RWC) was evaluated according to the formula: RWC (%) = (FW − DW)/(TW− DW) × 100. Here, FW is the fresh weight, TW means the turgid weight and DW delegated the dry weight.

### Determining MDA level and relative electrical conductivity

MDA (malondialdehyde) levels and relative electrical conductivity were estimated according to Li et al. [25].

### Histochemical ROS staining, assay of antioxidant enzyme activity

H_2_O_2_ (hydrogen peroxide) and O_2_^−^ (superoxide) accumulation were assessed using the DAB and NBT staining methods described by Kong et al. [26].

Tobacco leaves subjected to drought stress were collected to measure the activities of antioxidant enzymes, including superoxide dismutase (SOD), peroxidase (POD), catalase (CAT), ascorbate peroxidase (APX), and dehydroascorbate reductase (DHAR), as described previously [27–29]. The activity of monodehydroascorbate reductase (MDHAR) was determined according to Dat et al. [30]. The assay of enzyme activity was carried out using a spectrophotometer at 25°C.

### Statistical analysis

All experiments were repeated a minimum of three times. Data were analyzed using the software Excel and DPS. Statistical significance was tested using Duncan’s test at the 0.05 and 0.01 probability levels.

## Results

### The deduced cis-regulatory element in the *TaEXPA2* promoter

We obtained a 1073-bp promoter fragment upstream of the ATG translation initiation codon of *TaEXPA2* (S1 Fig) and analyzed its cis-regulatory elements using the PLACE [31] and PlantCARE databases [32–33]. Several regulators were found, including cis-acting elements involved in light responses, phytohormone signaling, and abiotic stress responsiveness, as well as an enhancer-like element involved in anoxic specific inducibility meristem expression, cell cycle regulation, zein metabolism regulation, seed-specific regulation, circadian control, and so on (Table 1). For instance, abscisic acid response elements (ABREs) were found for abscisic acid responses; the CGTCA-motif and TGACG-motif were found for MeJA responses; the GARE-motif was found for GA responses; and the MBS and GC-motifs were found for drought stress responses. These imply the possible involvement of *TaEXPA2* in plant development and stress tolerance.

**Table 1:** Putative cis-elements in the promoter region of *TaEXPA2* as predicted by PlantCARE.

Function	Cis-element	Position	Sequence (5’-3’)
Light responsive element	AE-box	-909(-)	AGAAACAT
	ATCT-motif	-426(-)	AATCTGATCG
	Box4	-676(+),-948(+)	ATTAAT
	G-box	-669(-)	CACGTA
		-177(-),-261(+),-703(-),	CACGAC
		-304(-),-408(+)	CACATGG
		-669(+)	TACGT
	GAG-motif	-114(-)	GGAGATG
	GT1-motif	-961(-)	GGTTAA
		-403(+)	GGTTAAT
	Sp1	-298(+)	GGGCGG
		-831(-)	CC(G/A)CCC
abscisic acid responsiveness	ABRE	-669(+)	TACGTG
MeJA-responsiveness	CGTCA-motif	-222(-)	CGTCA
	TGACG-motif	-222(+)	TGACG
gibberellin-responsive element	GARE-motif	-943(-)	AAACAGA
drought-inducibility	MBS	-509(+),-800(+)	TAACTG
		-255(-)	CAACTG
	GC-motif	-142(-)	CCCCCG
enhancer-like element involved in anoxic specific inducibility meristem expression	CAT-box	-970(-),-338(-)	AGTGGC
cell cycle regulation	MSA-like	-766(-)	TCCAACGGT (T/C)C (T/C)
		-61(+)	AACGG (T/C)(T/C)A
cis-acting regulatory element involved in zein metabolism regulation	O2-site	-411(-)	GATGACATGG
cis-acting regulatory element involved in seed-specific regulation	RY-element	-523(+)	CATGCATG
circadian control	circadian	-369(+)	CAANNNNATC

### Expression patterns of *TaEXPA2* in different organs of wheat and their responses to abiotic stresses and phytohormones

**Fig 1:**
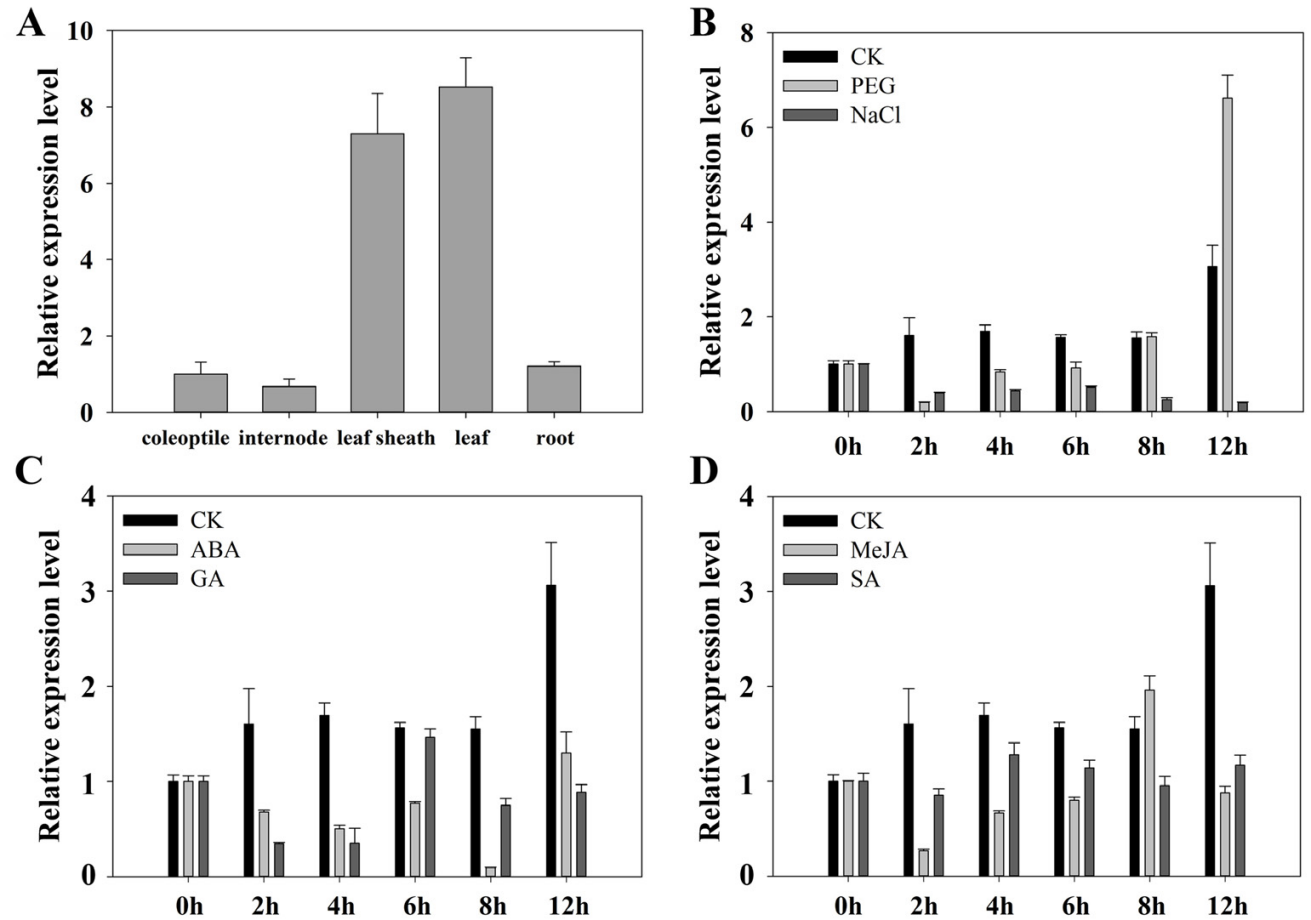
Expression profiles of *TaEXPA2* in different tissues of wheat plants in response to abiotic stresses and signaling molecules, as detected by qRT-PCR. (A) Expression of *TaEXPA2* in different organs/tissues of wheat. (B-D) Total RNA was isolated from 2-week-old wheat seedling leaves that were collected after exposure to 20% PEG and 150 mM NaCl (B), 2 mM ABA, 50 mM GA (C), 10 mM MeJA, and 10 mM SA (D). Wheat seedlings incubated in sterile water (CK) were used as controls. The α*-tubulin* gene was used as an internal reference. The experiments were repeated at least three times.

We performed qRT-PCR analysis to characterize the expression patterns of *TaEXPA2*. This gene was expressed in all of the analyzed organs, including the wheat coleoptile, internode, leaf sheath, leaf, and root (Fig 1A). The transcript levels were highest in leaf.

We then tested the expression levels in 2-week-old wheat seedlings exposed to PEG, NaCl, ABA, GA, MeJA, and SA. Although the transcription level of *TaEXPA2* was slightly reduced at first, it strongly increased after 12 h of PEG treatment compared to the control. Under the other treatments, the accumulation of *TaEXPA2* mRNA was down-regulated to various degrees (Fig 1B–1D). This suggests that *TaEXPA2* may be involved in the plants’ responses to various stresses through different signaling mechanisms.

### Subcellular localization of TaEXPA2

**Fig 2:**
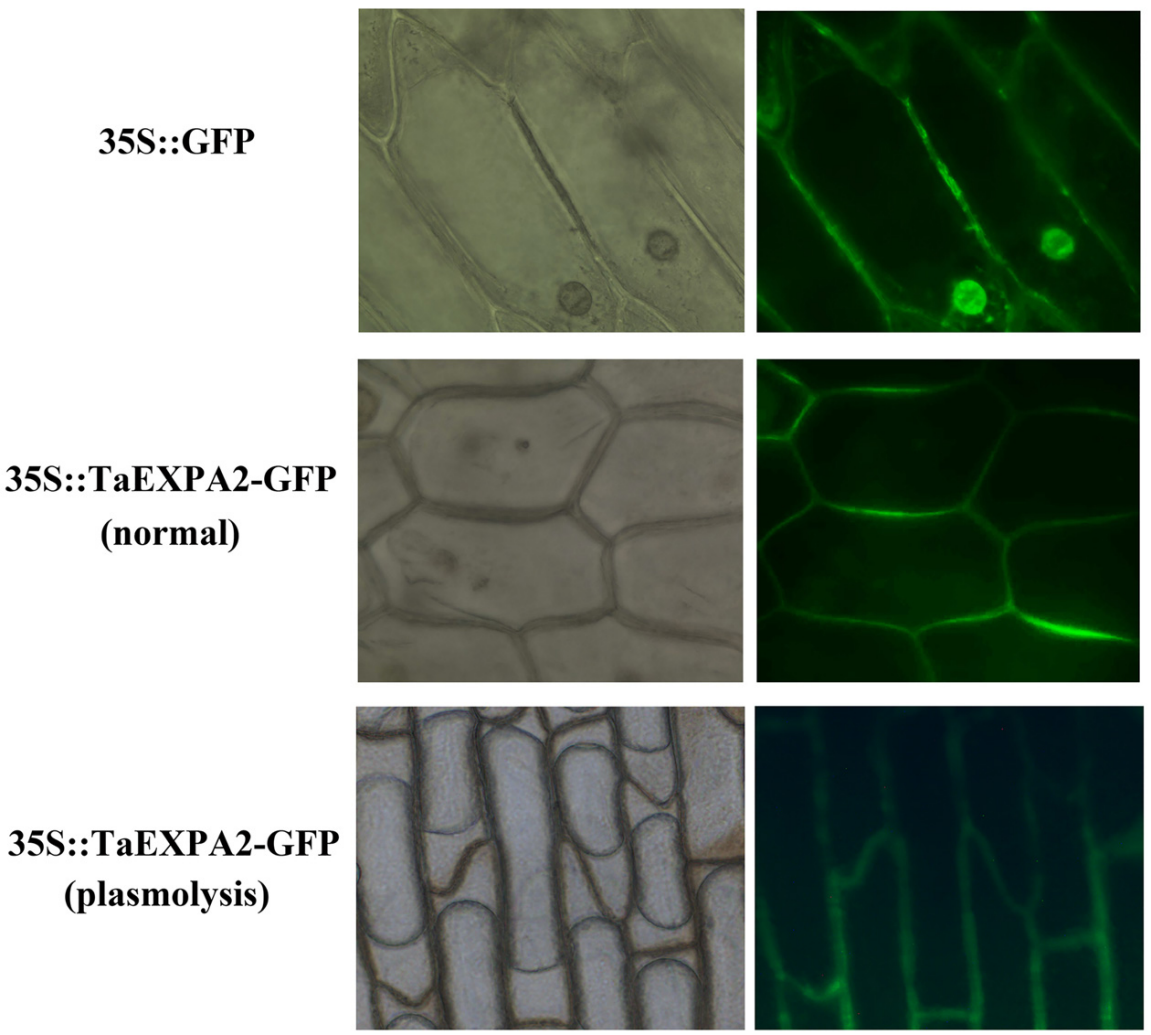
Subcellular localization of the TaEXPA2-GFP protein in onion epidermal cells. The transient expression of *35S*::*GFP* and *35S*::*TaEXPA2-GFP* in onion epidermal cells. The cells were analyzed by laser confocal microscopy after culture on MS medium at 28°C for 2 days. Onion cell plasmolysis was induced by a 30% sucrose solution for 20 min before observation.

To investigate the subcellular localization of the TaEXPA2 protein in plant cells, we performed transient expression assays with plasmids expressing green fluorescence protein (GFP) alone, and expressing the TaEXPA2-GFP fusion protein. Onion epidermal cells were used to transiently express the fusion protein through the *Agrobacterium*-mediated transformation method. The cells transformed with the *35S*::*GFP* control vector showed GFP signals in the entire cytoplasm and nucleus, whereas cells transformed with *35S*::*TaEXPA2-GFP* (with or without plasmolysis) exhibited green fluorescence only in the cell wall. These results confirmed that the TaEXPA2 protein is localized at the cell wall (Fig 2).

### The generation and identification of the transgenic tobacco plants

**Fig 3:**
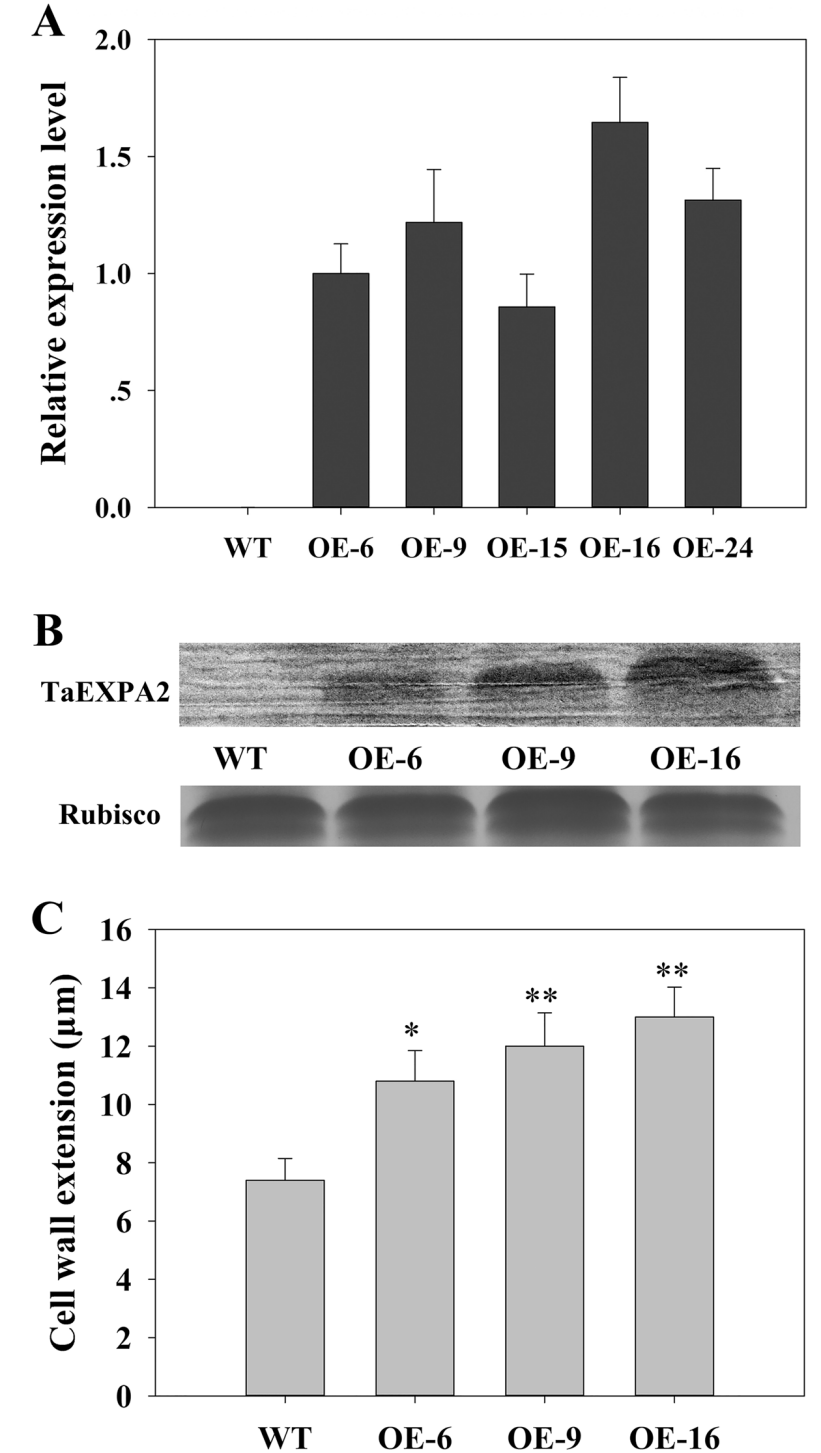
Confirmation of *TaEXPA2* transgenic tobacco plants. (A) The relative expression level of *TaEXPA2* was assessed by quantitative real-time PCR. The mRNA levels in the WT plants and five different *TaEXPA2* transgenic lines (OE-6, OE-9, OE-15, OE-16, and OE-24). (B) TaEXPA2 protein abundance in the cell walls of WT and transgenic tobacco leaves by immunoblot analysis. Cell wall protein extracts were prepared from growing leaves of transgenic and WT tobacco plants. RuBisCO large subunit (Agrisera, AS03037) was used as a loading control. (C) Expansin activity in different transgenic lines and WT plants. Expansin activity was assayed by measuring the increase in the extension rate of wheat coleoptiles after the addition of the cell wall extract from leaves of different tobacco lines. Data represent the mean values for the three independent biological replicates. Standard errors are indicated by vertical bars. * and ** indicate significant differences from the WT values at P < 0.05 and P < 0.01, respectively, according to Duncan’s multiple range test.

The full-length sequence of *TaEXPA2* was cloned into the plant expression vector, named pBI121, using the cauliflower mosaic virus 35S promoter. Then the vectors with *35S*::*TaEXPA2* were inserted into tobacco leaves. The transformants were detected by qRT-PCR analysis. Five independent homozygous lines showed high levels of *TaEXPA2* expression, whereas the WT plants exhibited no expression (Fig 3A). The expression of TaEXPA2 in the leaf cell wall of three of these transgenic lines (OE-6, OE-9, and OE-16), and its absence in the WT plants, was further confirmed by western blot analysis using an antibody raised against TaEXPA2 (Fig 3B). No bands were observed in WT plants, whereas clear positive bands were found in lanes containing transgenic plant extract. We also measured the activity levels of expansin in the cell walls of WT and transgenic lines. The transgenic plants consistently had 1.5-fold to 1.8-fold higher levels of expansin activity than the WT plants (Fig 3C). These results demonstrated that the *TaEXPA2* gene was successfully introduced into the tobacco plants and it substantially increased the activity of expansin.

### Seed production was increased in *TaEXPA2*-overexpressing tobacco plants

**Fig 4:**
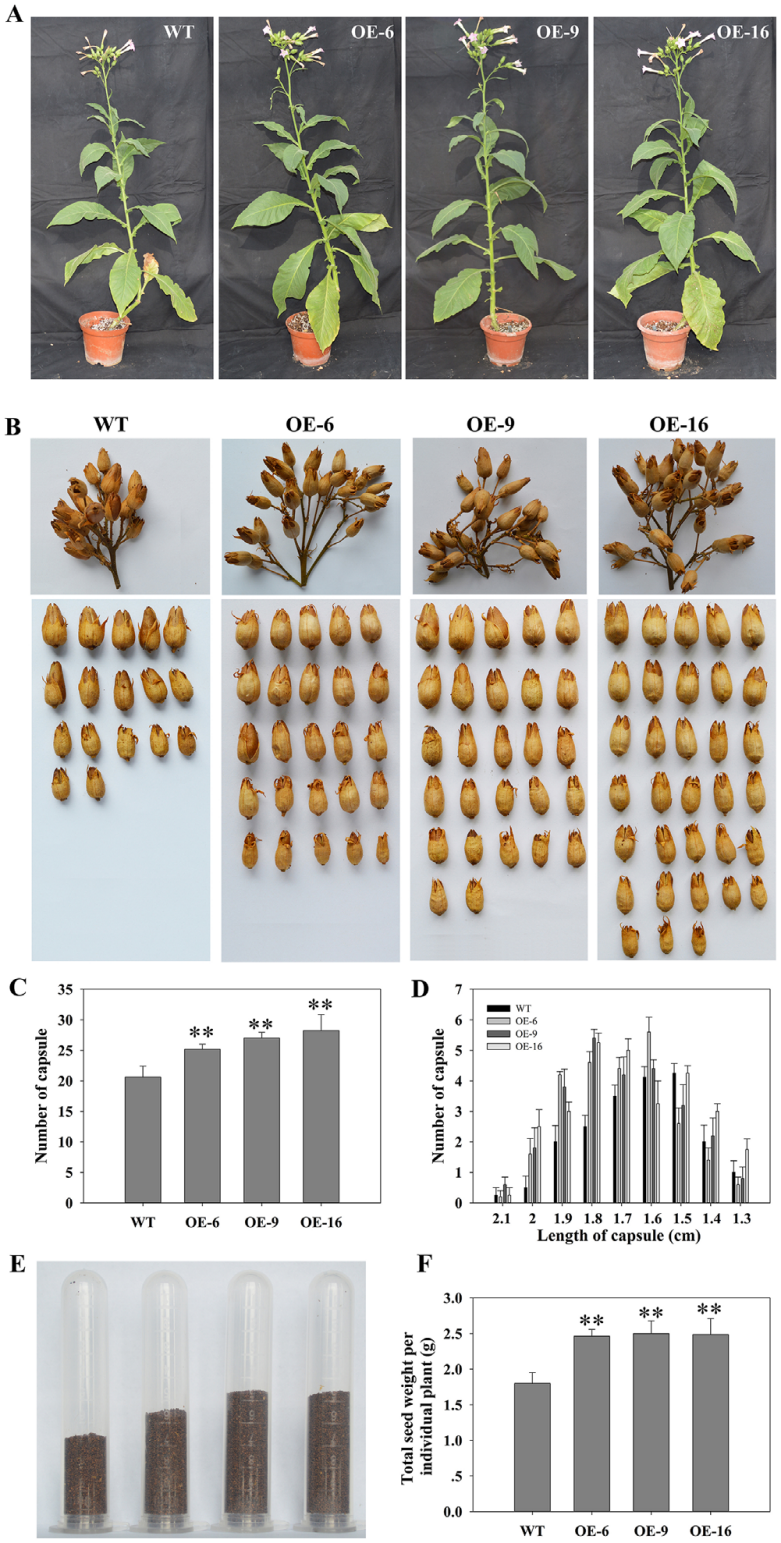
Seed production of WT and *TaEXPA2* transgenic lines. (A) Phenotypic comparison of WT and *TaEXPA2* transgenic lines in the flowering stage. (B) The spike and capsules. (C) Total number of capsules per tobacco plant. (D) Distribution statistics of capsule length. (E) Seed yield of individual tobacco plants. (F) Total seed weight per tobacco plant. Data in (C), (D), and (F) represent the mean values for the six independent biological replicates. In (C) and (F), * and ** indicate significant differences from the WT values at P < 0.05 and P < 0.01, respectively, according to Duncan’s multiple range test.

To examine the phenotypic changes caused by the overexpression of *TaEXPA2* in transgenic tobacco plants, we continued our investigations over the whole development process. Compared with WT plants, transgenic plants did not show any obvious alteration in growth patterns, including leaf number, leaf area, internode length, plant height, and anthesis. However, the transgenic plants were found to have a larger inflorescence with more capsules compared to WT plants (Fig 4A and 4B). Moreover, the number of capsules in the plants from each of the three transgenic tobacco lines corresponded, in order, to the relative *TaEXPA2* expression levels and expansin activity levels in those three transgenic lines (Fig 4C). When we examined capsule length, we found that WT plants had values primarily in the 1.5–1.7 cm range, whereas capsule length in the three transgenic tobacco lines was primarily in the 1.6–1.9 cm range (Fig 4D). Seed production per plant was also higher in the transgenic tobacco than in the WT plants (Fig 4E), and this was also supported by statistical data (Fig 4F).

**Fig 5:**
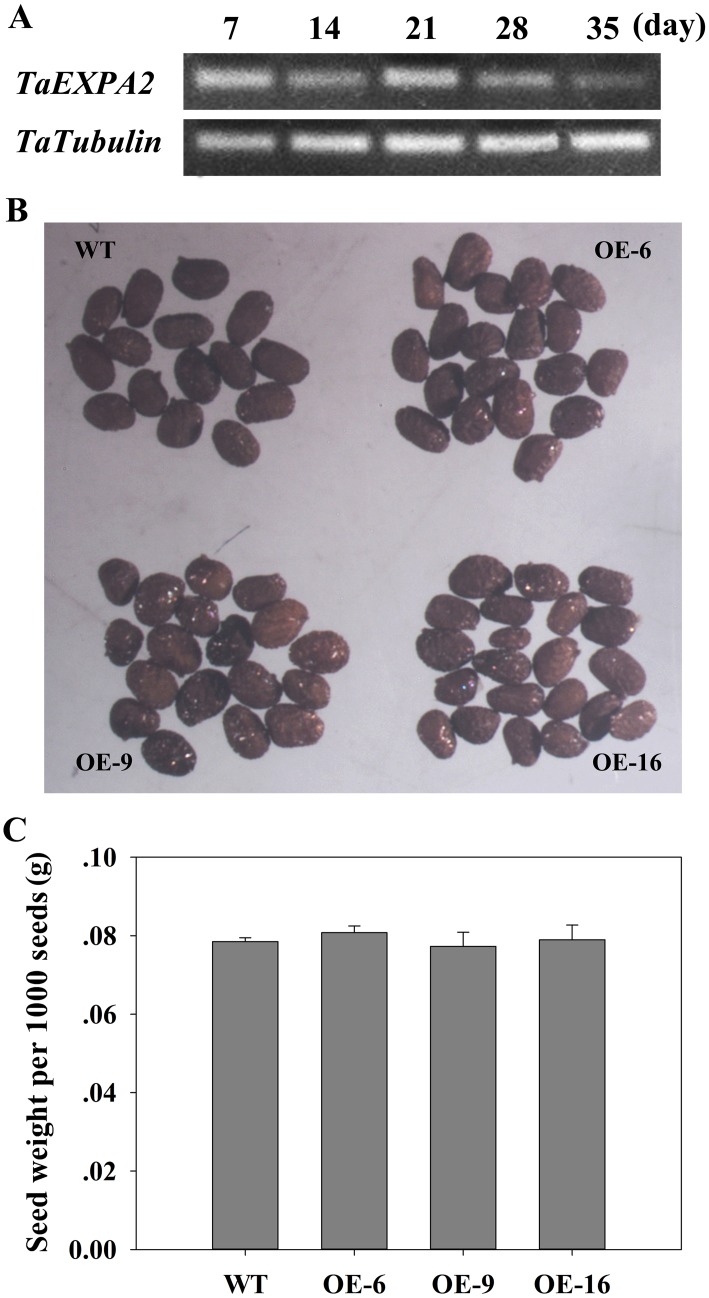
Seed size comparison and expression profile of *TaEXPA2* during wheat-grain-filling in WT and transgenic plants. (A) The level of *TaEXPA2* expression during wheat-grain-filling by RT-PCR. The wheat grains were collected after pollination at intervals of 7 days to isolate total RNA. *TaTubulin* was used as an internal control. (B) Comparison of seed size in WT and *TaEXPA2* transgenic tobacco plants. These images were obtained under the same magnification using a dissecting microscope (OLYMPUS SZX12). (C) Total weight of one thousand seeds. Data represent the mean values for the six independent biological replicates.

In particular, we detected the expression of *TaEXPA2* during wheat seed development. The expression level of *TaEXPA2* changed in wheat seeds during the grain-filling period, and was especially high at 21 days after grain fertilization (Fig 5A). However, the seed sizes of the transgenic tobacco plants were similar to the WT plants (Fig 5B). Moreover, no obvious difference was found in seed weight between WT and transgenic tobacco lines (Fig 5C). This was inconsistent with results found in *Arabidopsis* and cotton: in addition to an increase in the number of *Arabidopsis* siliques or cotton bolls, seed size was also larger in expansin-gene overexpressing lines than in WT plants [34–35].

### Overexpression of the *TaEXPA2* gene enhanced the tolerance of the transgenic plants to drought stress

**Fig 6:**
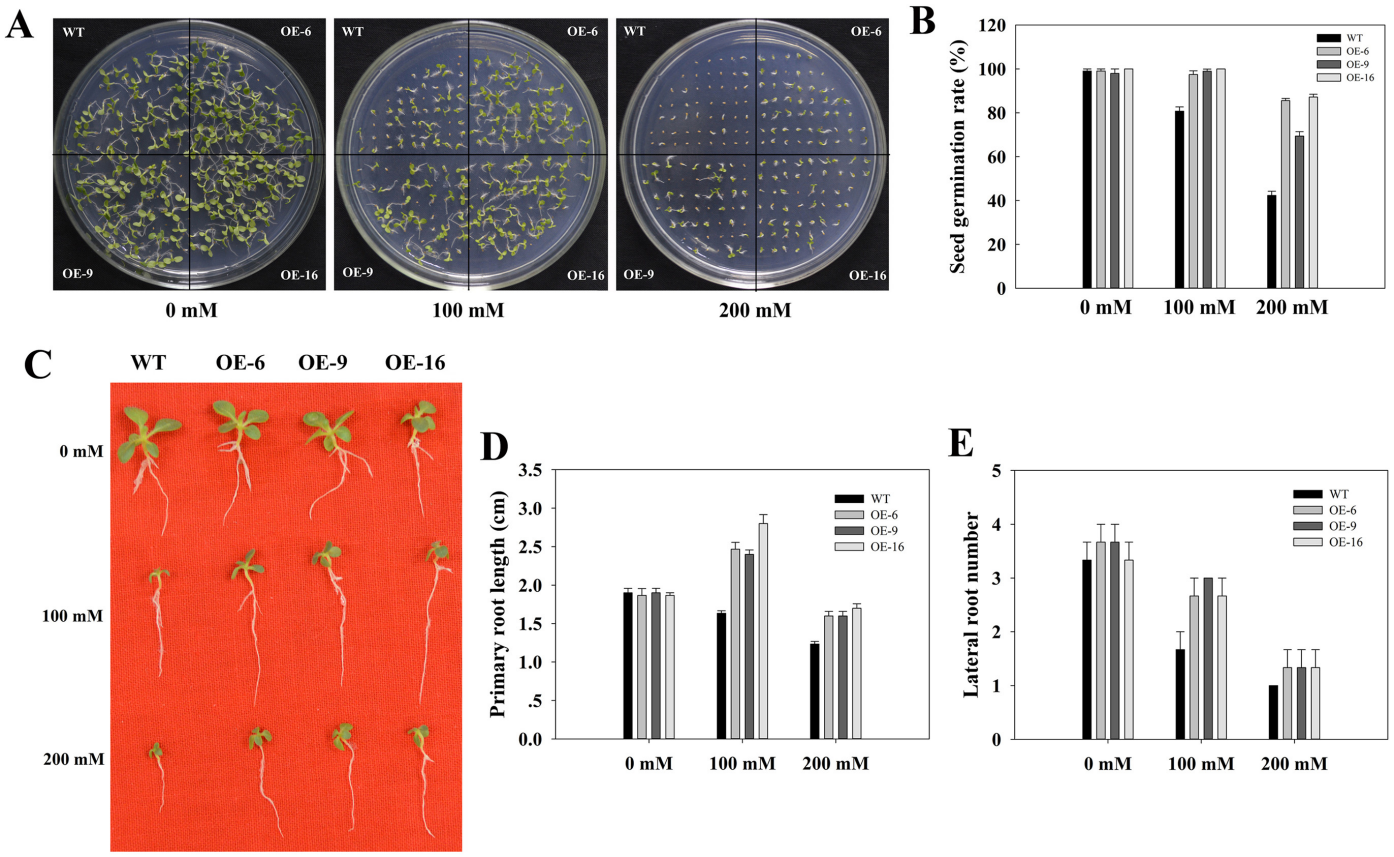
Seed germination and young seedling development in *TaEXPA2* transgenic lines and WT plants under water deficient stress. (A) The germination of WT and *TaEXPA2* transgenic tobacco seeds in the presence of 100 and 200 mM mannitol, and (B) the corresponding germination rate. The germination rate was counted 10 days after sowing. (C) Phenotypic analysis of 7-day-old WT and *TaEXPA2* transgenic seedlings in the presence of 100 or 200 mM mannitol for 20 days. Primary root length (D) and lateral root numbers (E) were recorded. Each column represents an average of three replicates, and bars indicate SEs.

We next examined the functions of *TaEXPA2* in drought stress tolerance using our transgenic tobacco lines. First, the seeds of WT plants and of three *TaEXPA2*-overexpressing lines (OE-6, OE-9, and OE-16) were germinated on MS plates supplemented with different concentrations of mannitol. On MS plates without mannitol, the *TaEXPA2*-overexpressing lines did not show any obvious differences during germination compared to WT plants. However, on medium containing 100 or 200 mM mannitol, the germination rates of the transgenic lines were significantly higher than those of the WT plants (Fig 6A and 6B).

We then tested the growth response of the tobacco seedlings to drought stress. There was no significant difference in the mannitol-free medium. However, when treated with 100 or 200 mM mannitol, the transgenic lines exhibited longer primary root length and more lateral roots compared to the WT plants (Fig 6C–6E). Notably, the primary root length of the three transgenic lines treated with 100 mM mannitol was significantly greater than those on MS plates without mannitol.

**Fig 7:**
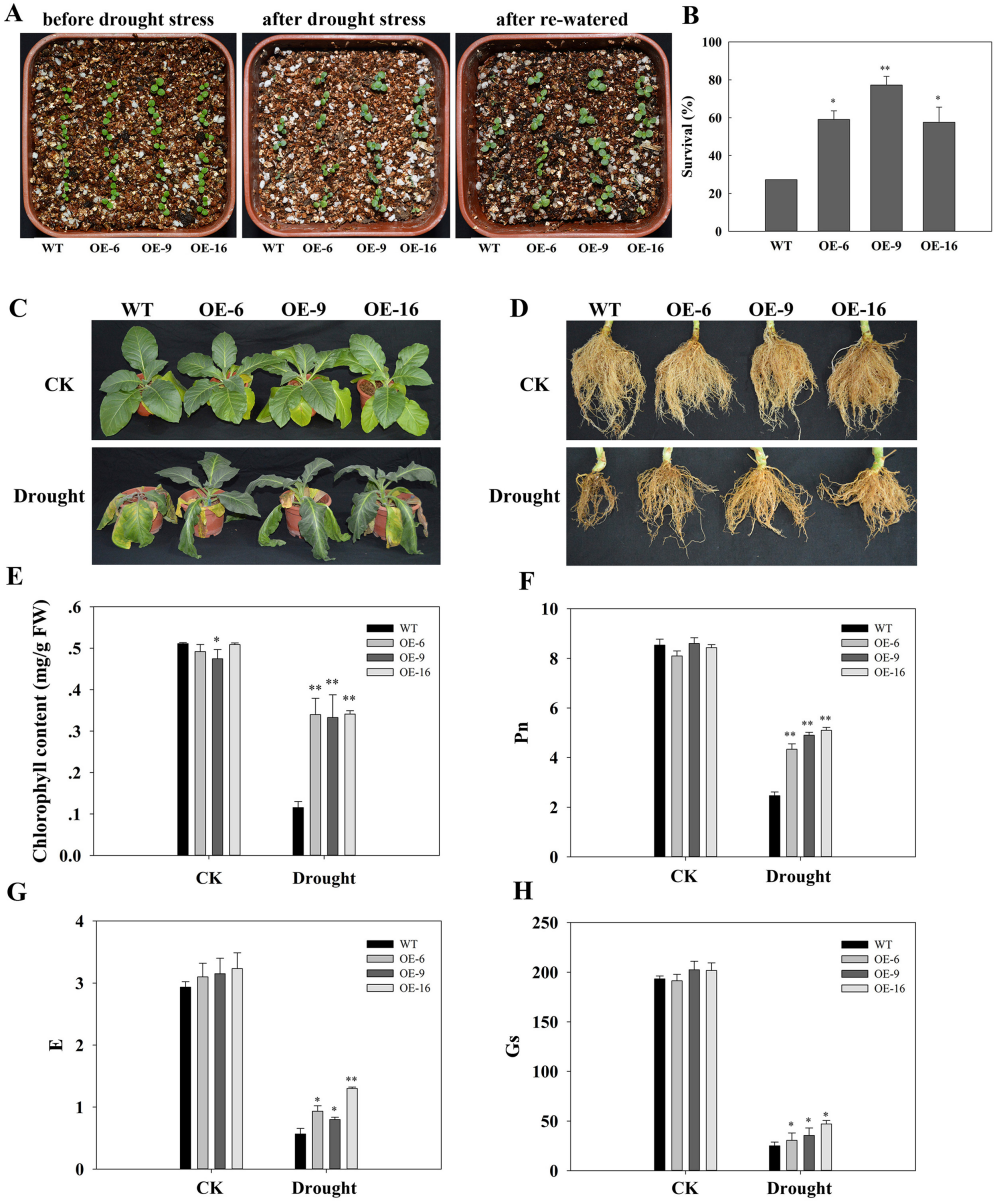
Evaluation of the drought tolerance of *TaEXPA2* transgenic tobacco plants. (A) 10-day-old WT and transgenic plants were exposed to natural drought stress for 7 days and re-watered for 2 days, and (B) the corresponding survival rate of transgenic plants after drought stress and re-watering. (C) 50-day-old WT and transgenic plants were naturally exposed to drought for 21 days with the well-watered plants as controls. (D) The corresponding root growth status from (C). (E) Chlorophyll content (mg/g FW). (F) Net photosynthetic rate (Pn, μmol CO_2_ m^-2^ s^-1^). (G) Transpiration rate (E, mmol H_2_O m^-2^ s^-1^). (H) Leaf stomatal conductance (Gs, mmol H_2_O m^-2^ s^-1^). The experiment was replicated three times, and bars indicate SEs. * and ** indicate significant differences of P < 0.05 and P < 0.01, respectively, according to Duncan’s multiple range test.

We also cultivated 10-day-old tobacco seedlings in an incubator, subjected then to drought stress naturally via water evaporation for 1 week, and then re-watered them for another 2 days (Fig 7A). The three transgenic lines showed higher survival rates than the WT plants (Fig 7B).

Next, 50-day-old transgenic and WT tobacco plants were cultivated on vermiculite under well-watered conditions and then were similarly subjected to natural water evaporation for 21 days (Fig 7C). After drought stress, the transgenic plants clearly showed more vigorous phenotypes than the WT plants, including more green leaves (Fig 5C) and denser survival roots (Fig 7D). The chlorophyll content of the transgenic lines was also higher, by approximately 3-fold, than it was in WT plants (Fig 7E). Drought stress caused a significant decline in the net photosynthetic rate (Pn), transpiration rate (E), and in leaf stomatal conductance (Gs) in both WT and transgenic plants (Fig 7F–7H). However, the photosynthetic ability (Pn) of WT plants was higher than that of transgenic plants. The Pn declined more rapidly in WT plants than in transgenic plants under drought stress (Fig 7F).

Based on the results shown in Figs 6 and 7, we suggest that overexpression of *TaEXPA2* improved the drought stress tolerance of transgenic tobacco plants.

### Overexpression of *TaEXPA2* enhanced the water retention capacity of transgenic tobacco leaves

**Fig 8:**
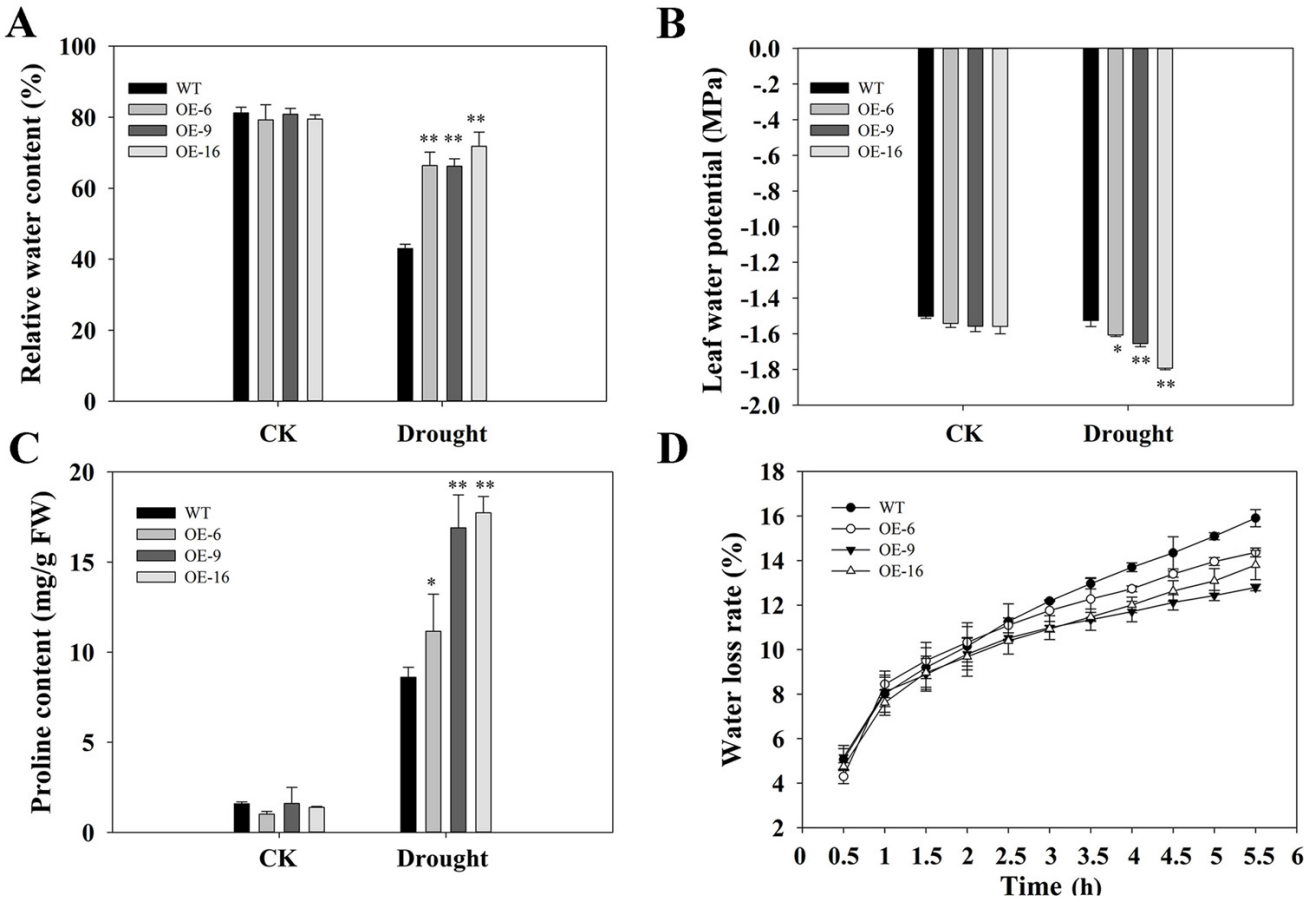
Evaluation of water loss, water potential, proline content, and kinetics of *TaEXPA2* transgenic tobacco plants. The corresponding relative water loss (A), water potential (B), and proline content (C) of the tobacco leaves undergoing water deficit stress shown in Fig 7. (D) The kinetics of water loss from the leaves of 50-day-old tobacco plants. Each column represents an average of three replicates, and bars indicate SEs. * and ** indicate significant differences at P < 0.05 and P < 0.01, respectively, according to Duncan’s multiple range test.

The RWC of tobacco leaves, shown in Fig 7C, demonstrated that transgenic plants can retain more water than WT plants under drought stress (Fig 8A). Moreover, *TaEXPA2*-overexpressing lines exhibited lower water potential than WT plants under water-insufficient conditions (Fig 8B). It is known that proline acts as an osmotic adjustment substance that protects plant cells under osmotic stress [36]. After drought stress, the proline content of all WT plants increased, but the levels were significantly higher in *TaEXPA2*-overexpressing plants than in WT plants (Fig 8C). In addition, when the tobacco leaves were dehydrated in identical environments, a steady decrease in fresh weight was observed in both the WT and transgenic lines. However, distinctly less water was lost in transgenic plants than in WT plants at all of the time points measured within 6 h of dehydration (Fig 8D), and this was in agreement with the trend seen in RWC (Fig 8A).

### Overexpression of *TaEXPA2* increased the antioxidant capacity of transgenic tobacco plants

It has been reported that abiotic stresses can injure cell membranes in plants by way of oxidative stress and the generation of reactive oxygen species (ROS) such as singlet oxygen, superoxide, and hydrogen peroxide [25–26]. Because the overexpression of *TaEXPA2* improved the drought stress tolerance of transgenic tobacco plants, we examined cell membrane damage and antioxidant capacity in our experimental lines.

**Fig 9:**
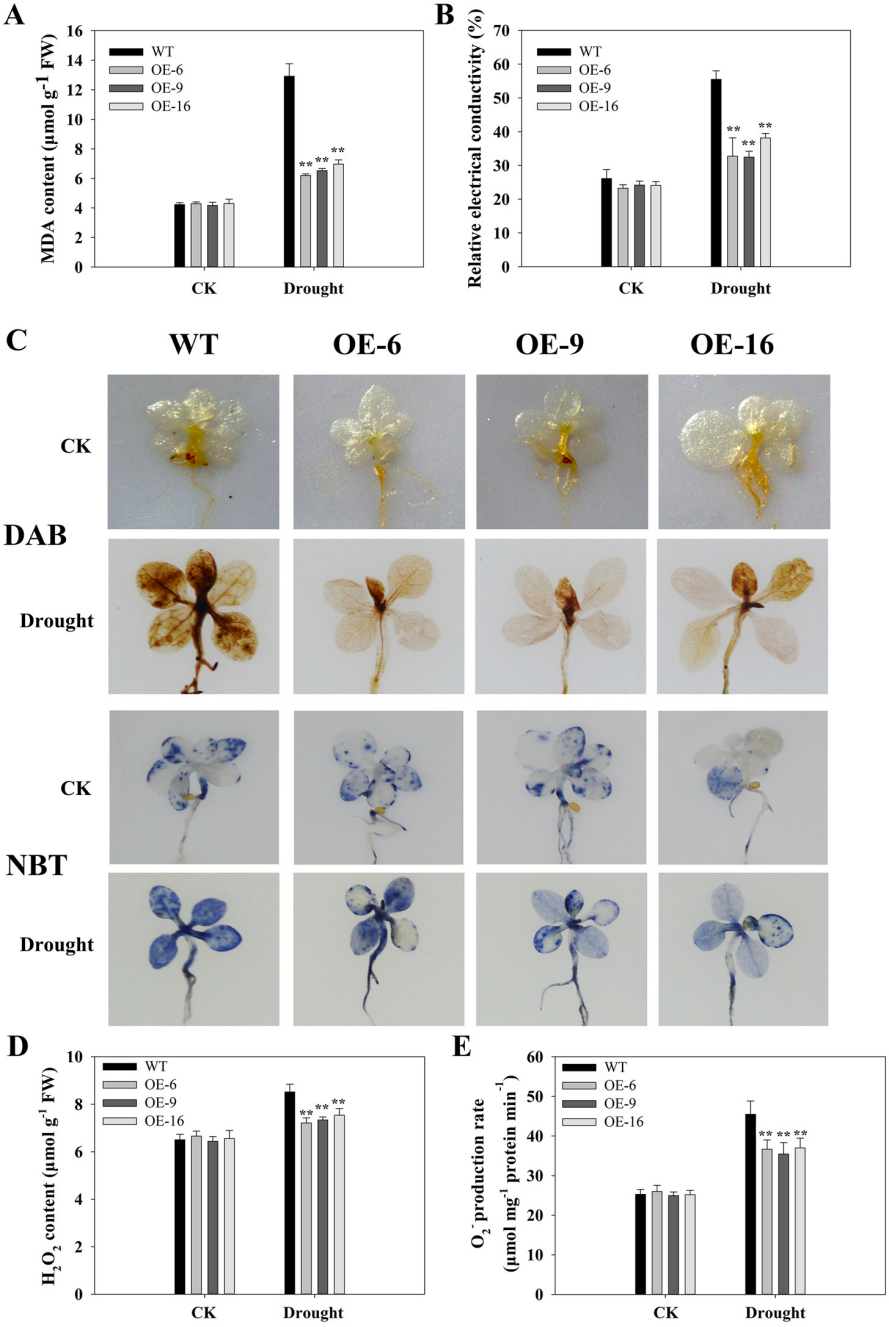
Oxidation conditions in WT and *TaEXPA2* transgenic plants under drought stress. Changes in MDA content (A) and relative electrical conductivity (B) in WT and transgenic tobacco leaves under drought stress. (C) In situ detection of H_2_O_2_ and O_2_^−^ by DAB staining (upper) and NBT staining (bottom) in WT and *TaEXPA2* transgenic seedlings grown on normal medium for 14 days and then treated with 100 mM mannitol for 7 days. The experiment was replicated three times. (D) H_2_O_2_ content and (E) O_2_^−^ production rate in 14-day-old plants treated with 100 mM mannitol for 7 days. Each column represents an average of three replicates, and bars indicate SEs. * and ** indicate significant differences of P < 0.05 and P < 0.01, respectively, according to Duncan’s multiple range test.

First, under well-watered control conditions, the MDA content of leaves from transgenic and WT plants were nearly equal, with the exception of the OE-16 plants, where the levels were slightly higher. After drought stress, the MDA content levels increased, but they increased less in *TaEXPA2*-overexpressing plants than in WT plants (Fig 9A). The relative electrical conductivity of the leaves in all plants was similar under control conditions (Fig 9B), but after drought stress it increased dramatically in all plants, and this increase was greater in WT plants than in transgenic plants.

Second, we determined the accumulation of H_2_O_2_ and superoxide (O_2_^−^) by 3,3′-diaminobenzidine (DAB) staining and nitroblue tetrazolium (NBT) coloration, respectively. The DAB staining assay showed different levels of H_2_O_2_ production in the seedlings of the WT and transgenic lines, and the brown precipitate accumulated in WT plants much more than in the transgenic lines. Similar results were observed for O_2_^−^ accumulation (Fig 9C). The H_2_O_2_ content (Fig 9D) and O_2_^−^ production rate (Fig 9E) increased after drought stress in all plants, but they were considerably higher in the WT plants.

**Fig 10:**
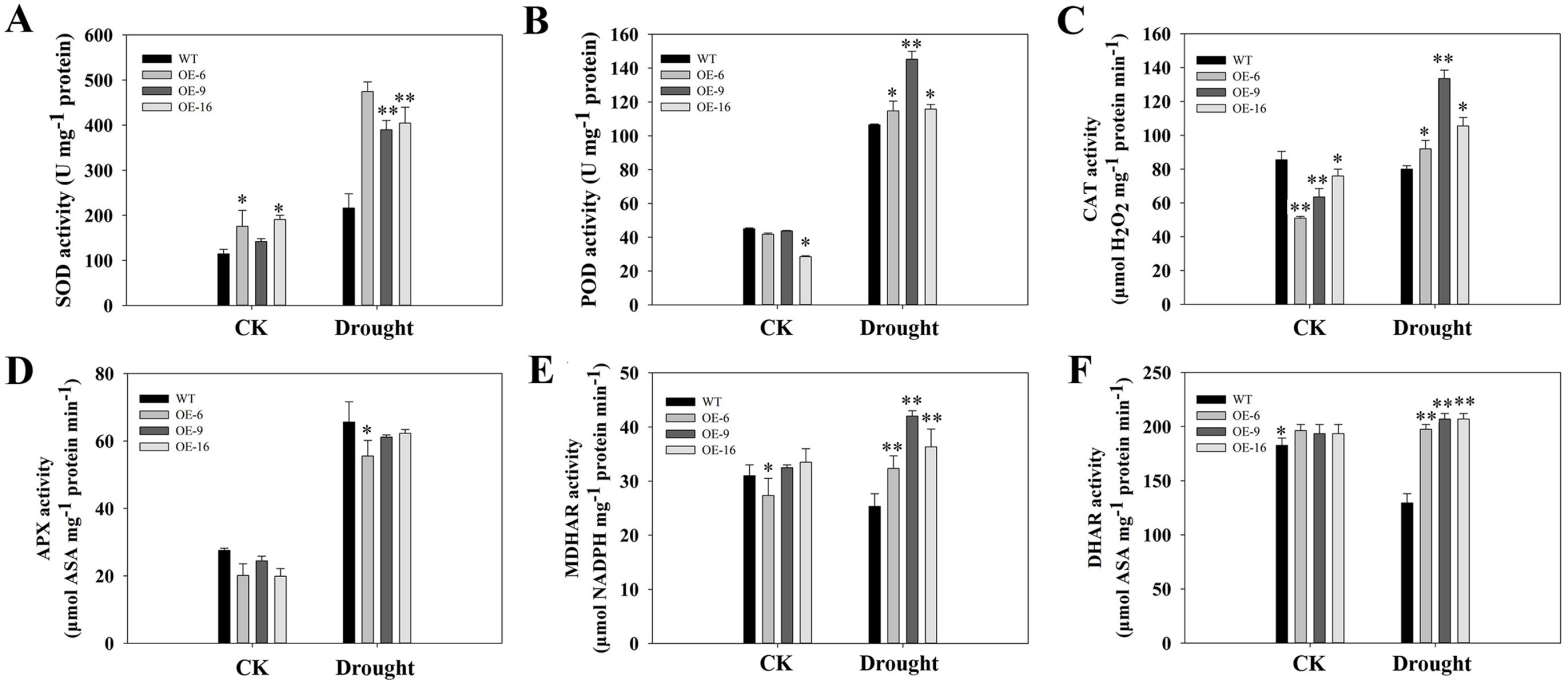
The activity levels of antioxidant enzymes in tobacco plants under drought stress. (A) Superoxide dismutase, SOD; (B) guaiacol peroxidase, POD; (C) catalase, CAT; (D) ascorbate peroxidase, APX; (E) monodehydroascorbate reductase, MDHAR; and (F) dehydroascorbate reductase, DHAR. Each column represents the mean ± standard error of three independent experiments. * and ** indicate significant differences from the WT values at of P < 0.05 and P < 0.01, respectively, according to Duncan’s multiple range test.

We next evaluated the activity of some antioxidant enzymes, including SOD, POD, CAT, APX, MDHAR, and DHAR. The results showed that SOD activity significantly increased after drought stress compared to normal conditions, and the increased activity of SOD in transgenic plants was greater than in WT plants (Fig 10A). Similar results were observed for the activity levels of POD and CAT (Fig 10B and 10C). The activity of APX was increased by drought stress treatment, but no obvious differences were found between the transgenic and WT plants (Fig 10D). The activity levels of MDHAR and DHAR in WT and transgenic plants were slightly different from those in the control. After drought stress, the activities of DHAR and MDHAR in WT plants were also reduced and were lower than in transgenic plants (Fig 10E and 10F).

**Fig 11:**
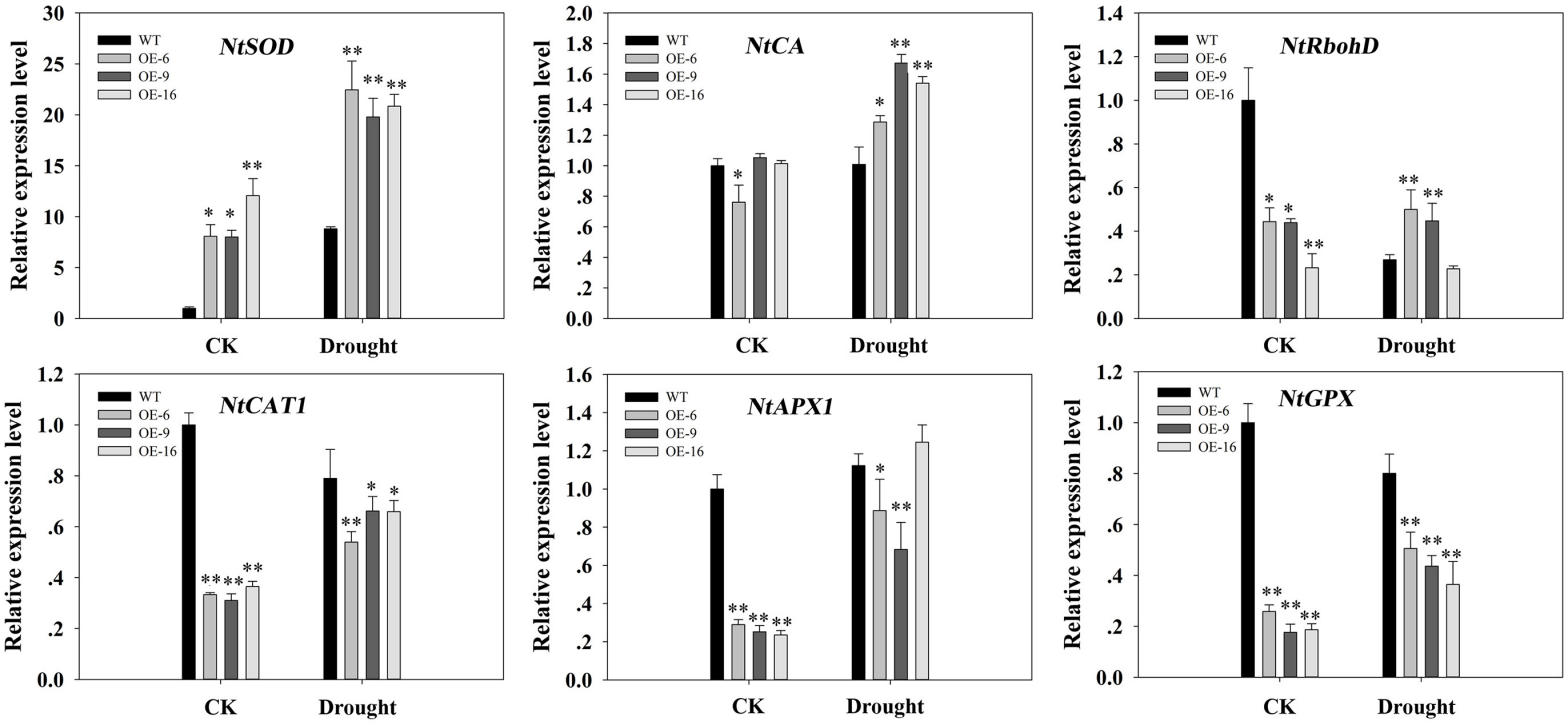
The expression of antioxidant-related genes in WT and transgenic tobacco plants under drought stress, as detected by qPCR. The transcript levels of these genes in transgenic plants are indicated relative to the levels in WT plants (taken as 1), and using transcripts of *actin* in the same samples as a reference. Each column represents the mean ± standard error of three replicates. * and ** indicate significant differences from the WT values at P < 0.05 and P < 0.01, respectively, according to Duncan’s multiple range test.

To analyze the molecular mechanisms behind the enhanced antioxidant enzyme activity in the transgenic tobacco, we performed a qPCR analysis on a set of known antioxidant-related genes in tobacco, including *NtSOD*, *NtCA*, *NtRbohD*, *NtCAT1*, *NtAPX1*, and *NtGPX*. Under well-watered conditions, the expression level of *NtSOD* was obviously higher in transgenic plants. Drought stress increased its mRNA accumulation, and the mRNA levels in the transgenic plants were much higher than those in the WT plants. No clear differences in *NtCA* expression between the WT and transgenic plants were observed without drought stress, but after drought stress the expression of *NtCA* in the transgenic lines increased, whereas in WT plants its expression level remained the same as the control. Compared to WT plants, the expression of *NtRbohD*, *NtCAT1*, *NtAPX1*, and *NtGPX* was slightly lower without drought stress. However, with the exception *NtRbohD*, the expression of all these genes was up-regulated slightly by drought stress (Fig 11).

## Discussion

Since the first expansin gene was discovered by McQueen-Mason et al. [7], an increasing number of expansin gene sequences have been cloned by different techniques and functionally characterized. Expansins are small proteins consisting of three regions, including an N-terminal signal peptide (attached to the cell wall), domain 1 (containing conserved Cys residues and an HFD motif), and domain 2 (a putative cellulose-binding domain). EXPAs have all of the aforementioned structural features, whereas in other expansin subfamilies, some of the domains may be missing. Notably, most EXPBs are predicted to have N-linked glycosylation sites near the amino and carboxyl termini, sites that EXPAs do not have [37]. This suggests that EXPAs and EXPBs have analogous but different functions.

Wheat is one of the most significant food crops worldwide. There are fewer functional studies of wheat expansins, however, because wheat is hexaploid, and this results in a large amount of genetic information [38]. In our previous work, we cloned a wheat EXPB gene named *TaEXPB23*. We found that *TaEXPB23* was involved in responses to abiotic stresses and phytohormone signals. Constitutive or root-specific overexpression of *TaEXPB23* enhanced the drought tolerance of transgenic tobacco lines [19,25,39]. The wheat gene in the present study, *TaEXPA2*, is an EXPA expansin gene. Liu et al. [16] detected a group of expansin gene expression patterns by real-time PCR and suggested that *TaEXPA2* plays a role in wheat cell wall growth. In this paper, we studied the gene’s functions in plant development and drought tolerance, and compared the similarities and differences between wheat α- and β-expansin genes.

### The mRNA transcription expression characteristics and subcellular localization of *TaEXPA2*

As the control region of a plant gene, a promoter will include multiple cis-regulatory elements that contribute to the complex expression profile of that particular gene. Thus, an analysis of promoter cis-regulatory elements is commonly used to investigate gene function [40–41]. In this study, we isolated the promoter fragment of *TaEXPA2* and predicted the putative cis-regulatory elements with PlantCARE. There are several types of cis-regulatory elements in the promoter fragment of *TaEXPA2*, and these are displayed in Table 1. Among these elements, we were especially interested in those related to phytohormone and abiotic stress responsiveness.

Different expansin clones were expressed in a variety of organ-specific patterns. For example, the *Festuca pratensis* α-expansins, *FpExp1*, *FpExp3*, *FpExp4*, and *FpExp5*, were only expressed in the 3 cm apical region of growing adventitious root tips. High levels of *FpExp2* mRNA were detectable in all four analyzed tissues, including the leaf elongation zone, coleoptile, apex, and roots. Transcripts of *FpExpB3*, a *Festuca pratensis* β-expansin, were low in the leaf elongation zone, coleoptiles, and root tips, but substantial accumulation was detected in the apex [42]. We measured the gene expression patterns of *TaEXPA2* in different wheat organs by qRT-PCR. *TaEXPA2* was expressed in all of the wheat organs examined, but the highest expression level was observed in leaves (Fig 1A).

Expansins can be induced by the classical defense hormones [43]. Based on the putative cis-regulatory elements in Table 1, some stress response and signaling elements were observed in the promoter of *TaEXPA2*. We evaluated the responses of *TaEXPA2* expression to PEG, NaCl, ABA, GA, MeJA, and SA treatments. As shown in Fig 1B–1D, *TaEXPA2* expression responded to all of the stress treatments and phytohormone signaling molecules. The most obvious increase in *TaEXPA2* expression was caused by PEG-induced water stress (Fig 1B), indicating that *TaEXPA2* may be involved in plant drought tolerance.

Expansins are cell wall proteins that are implicated in the control of plant growth via loosening of the extracellular matrix [8]. Choi et al. [37] suggested that expansins are primarily distributed over the expanding cell wall and bind to the cell wall. However, there are at least three expansin proteins that, whereas possessing an N-terminal signal peptide for targeting to the cell wall, are known to be located in the plasma membrane: OsEXPA17, AtEXP7, HvEXPB7 [44–45]. In our study, we fused TaEXPA2 to GFP, and the subcellular localization of GFP-tagged TaEXPA2 protein in transiently transformed onion epidermal cells indicated that *TaEXPA2* encodes a cell wall protein (Fig 2).

### Overexpression of *TaEXPA2* increased seed yield of transgenic tobacco by increasing capsule number, not seed size

By detecting the expression levels of *TaEXPA2*, as well as TaEXPA2 protein accumulation in leaf cell walls and the activity levels of expansin, we confirmed that *TaEXPA2* was overexpressed in the transgenic tobacco plants (Fig 3). Three transgenic lines, OE-6, OE-9, and OE-16, were selected to investigate the functions of *TaEXPA2*.

Expansin is involved in a variety of plant growth and development processes, such as vegetative and reproductive growth and fruit development [37]. With respect to seed production, two *GmEXPB2*-overexpressing transgenic soybean lines showed improved yield potential under low P conditions [46]. Overexpression of the sweet potato expansin gene *IbEXP1* in *Arabidopsis* increased the number of rosette leaves and siliques, as well as the seed sizes, a process that may be regulated by brassinosteroid (BR) signaling [35]. Xu et al. [34] also found that overexpression of the cotton α-expansin gene *GhEXPA1* in either *Arabidopsis* or cotton led to a substantial increase in the number of siliques or bolls, and an increase in leaves and seed sizes, leading to higher seed production. In this study, we found that transgenic tobacco had more capsules and higher seed production, even though the seed size itself was similar between the WT and transgenic tobacco plants (Figs 4 and 5) and there were no significant changes in vegetative and reproductive growth patterns.

### Overexpression of *TaEXPA2* enhanced the drought tolerance of the transgenic tobacco plants

Because the most obvious increase in *TaEXPA2* expression resulted from PEG-induced water stress (Fig 1B), we examined the involvement of *TaEXPA2* in plant drought tolerance. We observed that overexpressing *TaEXPA2* confers water stress tolerance on transgenic tobacco seedlings by means of higher germination rates and longer primary roots, as well as more lateral roots under water stress (Fig 6). Moreover, the higher survival rate and improved growth phenotype indicated that the drought tolerance of the transgenic tobacco plants was superior to that of WT plants (Fig 7A–7D). The photosynthetic parameters shown in Fig 7E–7G demonstrate that the transgenic plants maintained higher chlorophyll content, Pn, E, and Gs under drought stress conditions than the WT plants, and this finding suggests an improved drought tolerance in the transgenic plants compared with the WT plants. These results support the hypothesis that overexpression of *TaEXPA2* enhanced the drought tolerance of the transgenic tobacco plants.

### Osmotic adjustment and a larger root system, as well as enhanced oxidative competence, conferred drought tolerance on the transgenic tobacco plants

Plants resist drought by several means, including: (a) escaping drought by having a short life cycle or developmental adaptability; (b) avoiding drought through improved water uptake and reduced water loss; and (c) tolerating drought through osmotic adjustment, antioxidant capacity, and aridness tolerance [47]. Our results suggest that several mechanisms are involved in improving drought resistance in *TaEXPA2*-overexpressing transgenic tobacco plants.

First, the observed higher germination rate is evidence of developmental plasticity (Fig 6A and 6B). Under drought stress, earlier complete germination is beneficial to plants in that it allows them to escape from adverse environments.

Second, the larger root system of the transgenic plants (Figs 6C–6E and 7D) may take up more water under finite water conditions and so boost drought avoidance. Plant root systems are critical organs that are necessary for acquiring water and nutrients, for responding to abiotic signals derived from the soil, and for allowing plants to survive in different ecological niches [48]. Water absorption is the central function of the root system and it occurs primarily through root hairs, which, as a result of their density, constitute a large absorption surface [49]. After drought stress, the survival roots of transgenic lines were more extensive than those of WT plants (Fig 7D).

Third, the overexpression of *TaEXPPA2* enhanced the drought tolerance of the transgenic tobacco plants in at least two respects: osmotic adjustment and antioxidant capacity. The transgenic plants had higher relative water content and lower water potential values than WT plants (Fig 8A and 8B). Proline, as an osmotic adjustment component, plays an important role in plant adaptation to stresses caused by various environmental stimuli [36]. Under osmotic stress conditions, the proline content of *TaEXPA2*-overexpressing plants was evidently higher than in WT plants (Fig 8C). In addition, less water was clearly lost in transgenic plants than in WT plants at all of the time points measured within 6 h of dehydration (Fig 8D). With regard to the apparent disagreement between the lower water loss rate in transgenic plants shown in Fig 8D, and the higher transpiration rate (E) and Gs shown in Fig 7G and 7H, we suggest that transgenic plants can absorb more water through their larger roots (Fig 7D) and can also hold more water by osmotic adjustment under water stress conditions, even considering the greater Gs and E.

By contrast, drought stress leads to the overproduction of ROS. ROS have an adverse effect on cell growth by oxidizing lipids, proteins, and DNA [50]. There is ample evidence that antioxidative systems are involved in plant drought tolerance [25,51]. Levels of the cytomembrane lipid peroxidation product MDA are currently measured to indicate lipid peroxidation levels [52]. Compared to WT plants, the MDA content in transgenic plants was lower (Fig 9A). Similar results were observed in relative electrical conductivity (Fig 9B). Furthermore, in *TaEXPA2*-overexpressing tobacco, H_2_O_2_ and O_2_^−^ were present at low levels under drought stress, as revealed by DAB and NBT staining (Fig 9C–9E). The low level of ROS in the transgenic tobacco plants was related to the high activity of antioxidant enzymes such as SOD (Fig 10A), POD (Fig 10B), and CAT (Fig 10C) under drought stress conditions, with APX (Fig 10D) as an exception. Ascorbate peroxidase (APX), together with monodehydroascorbate reductase (MDHAR) and dehydroascorbate reductase (DHAR), removes H_2_O_2_ through the Halliwell-Asada pathway [53]. The activity levels of MDHAR and DHAR were also higher in the transgenic plants than in the WT plants (Fig 8E and 8F).

We also examined the expression levels of some antioxidant-related genes in transgenic plants, including *NtSOD*, *NtCA*, *NtRbohD*, *NtCAT1*, *NtAPX1*, and *NtGPX*. The expression levels of some of these genes changed in a variety of ways. Notably, the expression level of *NtSOD* dramatically increased, consistent with the SOD activity, suggesting that *NtSOD* may play an important role in the improved antioxidative systems of the transgenic tobacco plants (Fig 11).

### Similarities and differences in the functions of the wheat α-expansin gene *TaEXPA2* and the β-expansin gene *TaEXPB23*

With respect to the functions of *TaEXPB23* and *TaEXPA2*, there are at least three similarities. First, they are both cell wall proteins and are located in the cell wall (Fig 2) [39]. Second, the overexpression of both of them improves drought stress tolerance in transgenic tobacco, and this occurs by similar mechanisms in both cases [19]. For instance, *TaEXPA2*-overexpressing tobacco plants developed more extensive survival roots under drought stress (Fig 7), and root-specific expression of *TaEXPB23* similarly produced a larger root system [25]. Third, compared with the WT plants, no significant changes were observed in the seed size of the transgenic tobacco plants when they were overexpressing either *TaEXPB23* or *TaEXPA2* (Fig 5B, S2 Fig).

We also noted differences between *TaEXPB23*- and *TaEXPA2*-overexpressing tobacco plants. First, their mRNA transcription expression characteristics in wheat are not the same. The highest *TaEXPA2* expression was in leaf and was induced by drought stress, whereas *TaEXPB23* exhibited its highest transcription level in wheat coleoptiles and was up-regulated by MeJA and salt, not drought stress [19,39]. Second, the phenotype of the two kinds of transgenic tobacco was different, with *TaEXPA2*-overexpressing lines increasing seed production and the number of capsules, but with unchanged developmental patterns, whereas *TaEXPB23* overexpression resulted in evident phenotypic alterations, such as advanced jointing and flowering stages, larger leaves, and a longer internode length [54].

## Conclusion

In summary, our results suggest that overexpressing the α-expansin gene *TaEXPA2* increases seed production without changing the developmental patterns of the transgenic tobacco plants. In addition, overexpression of *TaEXPA2* enhances the drought tolerance of the transgenic tobacco plants by enlarging the root system, improving osmotic adjustment, and enhancing oxidative competence.

## Supporting Information

S1 Fig Promoter sequence of *TaEXPA2*. Yellow mark: translation initiation codon. Red mark: transcription start site. Green mark: typical TATA-box of promoter.
(TIF)

S2 Fig Mature seed phenotype of WT and three *TaEXPB23* overexpressing tobacco lines (T3-1, T3-8, and T3-10). The components of this figure were obtained under the same magnification using a dissecting microscope (OLYMPUS SZX12).
(TIF)

S1 Table Primer names and sequences. (DOC)
