# Supplementary figures and images for: Overexpression of the Wheat Expansin Gene TaEXPA2 Improved Seed Production and Drought Tolerance in Transgenic Tobacco Plants
Source: PLoS One. 2016 Apr 13;11(4):e0153494. doi: 10.1371/journal.pone.0153494 (PMC4830583; doi:10.1371/journal.pone.0153494)

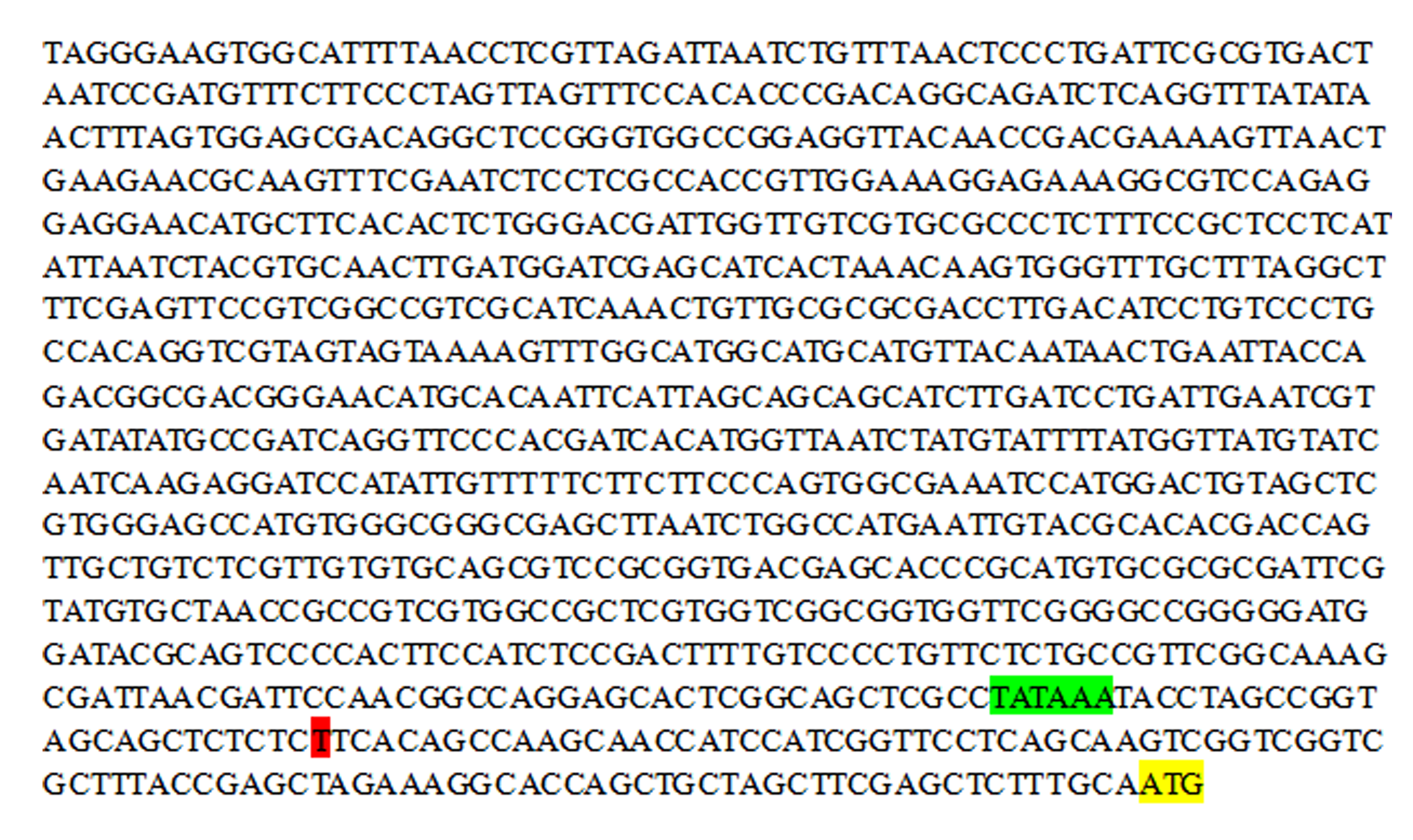

Supplement: S1 Fig — Yellow mark: translation initiation codon. Red mark: transcription start site. Green mark: typical TATA-box of promoter. (TIF) [file pone.0153494.s001.tif]

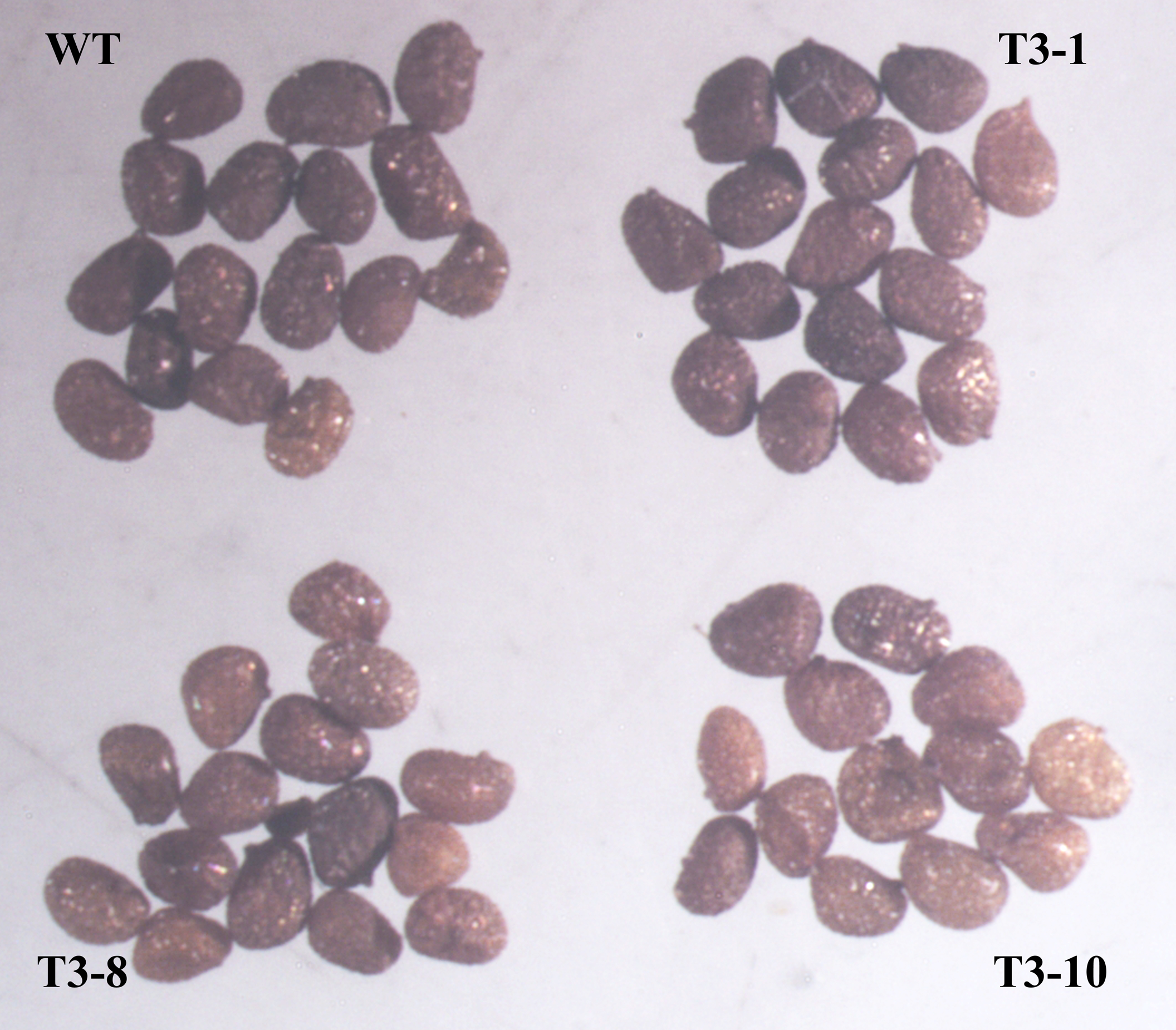

Supplement: S2 Fig — The components of this figure were obtained under the same magnification using a dissecting microscope (OLYMPUS SZX12). (TIF) [file pone.0153494.s002.tif]
