# Supplementary material for: Overexpression of the Wheat Expansin Gene TaEXPA2 Improved Seed Production and Drought Tolerance in Transgenic Tobacco Plants
Source: PLoS One. 2016 Apr 13;11(4):e0153494. doi: 10.1371/journal.pone.0153494 (PMC4830583; doi:10.1371/journal.pone.0153494)
Supplement: S1 Table — (DOC) [file pone.0153494.s003.doc]

**S1 Table**

Primer names and sequences.

| Name | Sequence (5’-3’) | Length (bp) |
| --- | --- | --- |
| *Gsp1* | ATGCAATCCATCCGTCCA | 18 |
| *Gsp2* | TCCCACCCTTCTTCACGCAC | 20 |
| *Gsp3* | TGGTGGCGGTAATGGTGATG | 20 |
| *AP1* | NTCGASTWTSGWTT | 14 |
| *AP2* | NGTCGASWGANAWGAA | 16 |
| *AP3* | WGTGNAGWANCANAGA | 16 |
| *AP4* | TGWGNAGSANCASAGA | 16 |
| *AP5* | AGWGNAGWANCAWAGG | 16 |
| *AP6* | STTGNTASTNCTNTGC | 16 |
| *A2-F* | GGATCCGGTCGCTTTACCGAGCTAGA | 26 |
| *A2-R* | GTCGACCAATGCAATCCATCCGTCCA | 26 |
| *GFP-1* | GTCGACGGTCGCTTTACCGAGCTAGA | 26 |
| *GFP-2* | GGATCCGAACTGGGCTCCTTCGA | 23 |
| *RTA2-F* | ACCGTCACCAGCAACAAC | 18 |
| *RTA2-R* | ATGCAATCCATCCGTCCA | 18 |
| *Tubulin-1* | ATCTGTGCCTTGACCGTATCAGG | 23 |
| *Tubulin-2* | GACATCAACATTCAGGACACCATC | 24 |
| *NtACTIN-F* | CATTGGCGCTGAGAGATTCC | 20 |
| *NtACTIN-R* | GCAGCTTCCATTCCGATCA | 19 |
| *NtSOD-F* | GACGGACCTTAGCAACAGG | 19 |
| *NtSOD-R* | CTGTAAGTAGTATGCATGTTC | 21 |
| *NtCA-F* | CGCCTGTGGAGGTATCAAA | 19 |
| *NtCA-R* | GAGAAGGAGAAAGACCGAACT | 21 |
| *NtRbohD-F* | ACCAGCACTGACCAAAGAA | 19 |
| *NtRbohD-R* | TAGCATCACAACCACAACTA | 20 |
| *NtCAT1-F* | TGGATCTCATACTGGTCTCA | 20 |
| *NtCAT1-R* | TTCCATTGTTTCAGTCATTCA | 21 |
| *NtAPX1-F* | GAGAAATATGCTGCGGATGA | 20 |
| *NtAPX1-R* | CGTCTAATAACAGCTGCCAA | 20 |
| *NtGPX-F* | GGTTTGCACTCGCTTCAAG | 19 |
| *NtGPX-R* | AGTAGTGGCAAAACAGGAAG | 20 |
